# In search of the pH limit of growth in halo‐alkaliphilic cyanobacteria

**DOI:** 10.1111/1758-2229.13323

**Published:** 2024-08-11

**Authors:** Lianchun Yi, Ruchita Solanki, Marc Strous

**Affiliations:** ^1^ Department of Earth, Energy, and Environment University of Calgary Calgary Alberta Canada

## Abstract

Cyanobacteria have many biotechnological applications. Increasing their cultivation pH can assist in capturing carbon dioxide and avoiding invasion by other organisms. However, alkaline media may have adverse effects on cyanobacteria, such as reducing the Carbon‐Concentrating Mechanism's efficiency. Here, we cultivated two halo‐alkaliphilic cyanobacteria consortia in chemostats at pH 10.2–11.4. One consortium was dominated by Ca. *Sodalinema alkaliphilum,* the other by a species of *Nodosilinea.* These two cyanobacteria dominate natural communities in Canadian and Asian alkaline soda lakes. We show that increasing the pH decreased biomass yield. This decrease was caused, in part, by a dramatic increase in carbon transfer to heterotrophs. At pH 11.4, cyanobacterial growth became limited by bicarbonate uptake, which was mainly ATP dependent. In parallel, the higher the pH, the more sensitive cyanobacteria became to light, resulting in photoinhibition and upregulation of DNA repair systems.

## Graphical abstract

Two halo‐alkaliphilic cyanobacteria consortia were cultivated in chemostats at pH 10.2–11.4. Increasing the pH decreased biomass yield. At higher pH levels, cyanobacteria were more sensitive to light and carbon might be transferred from the cyanobacteria to heterotrophs.

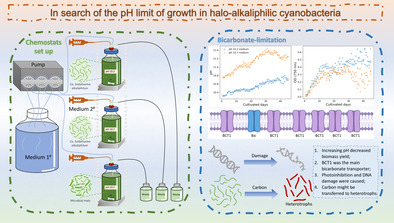

## INTRODUCTION

Cyanobacteria use sunlight for photosynthesis, assimilating carbon dioxide (CO_2_) into biomass. They have many biotechnological applications, such as producing nutrient supplements, fertilizers, pharmaceuticals and enzymes. Cyanobacteria are usually cultivated at a pH of 9–10 (Gao et al., 2023; Haines et al., 2022; Koch et al., 2022; Madkour et al., 2012; Raoof et al., 2006; Soni et al., 2019). Many studies have proposed that increasing pH and alkalinity can be advantageous to cyanobacterial biotechnology. This could both reduce the risk of production losses caused by invading microorganisms (Guidi et al., 2021) and improve CO_2_ supply or even enable the capturing of CO_2_ directly from the air (Ataeian et al., 2019; Zhu et al., 2020).

At pH 10–11, the CO_2_ concentration is low, but bicarbonate is still readily available (Tosca & Tutolo, 2023). To assimilate bicarbonate, cyanobacteria have evolved the carbon‐concentrating mechanism (CCM). The CCM combines active transport of bicarbonate (HCO_3_^−^) into the cytoplasm with converting HCO_3_^−^ to CO_2_ inside carboxysomes, protein microcompartments that contain RuBisCo. This leads to the intracellular production of hydroxide ions (OH^−^), which are removed from the cells, increasing the extracellular pH. The CCM comprises three bicarbonate transporters. One of these is powered by ATP hydrolysis (BCT1, CmpABCD, Omata et al., 2002) and the other two are sodium symporters (Sbt, Bic, Omata et al., 1999; Price et al., 2004). The energy costs associated with HCO_3_^−^ import may lead to significant (10%–33%) losses in biomass yield, reducing economic feasibility.

At some point above pH 11, the HCO_3_^−^ concentration will start to limit growth. At what pH this happens exactly is currently unknown, but this pH can be expected to be dependent on the total salt concentration (Millero et al., 2006) and the affinity of the bicarbonate transporters. Once, CO_2_ delivery to carboxysomes falters, RuBisCo may start to consume O_2_ instead of CO_2_ (photorespiration). For every two molecules of oxygen (O_2_) converted by RuBisCo, one previously assimilated carbon atom (C) is lost, further reducing economic feasibility.

Another potential energy expenditure induced by high pH is a higher cost associated with obtaining trace elements. Metal solubility generally demonstrates an inverse relationship with pH. As the pH of the medium increases, the solubility of many essential trace metals, especially iron, manganese and cobalt decreases (Butler, 1998). Finally, production or uptake of compatible solutes may also increase the overall energy costs (Dilworth & Glenn, 2007) and reduce biomass yield.

Several studies have shown the growth of cyanobacteria at high pH. For *Cyanobacterium* sp. PNNL‐SSL1 (Gao et al., 2023), the growth rate at pH 11.2 was lower compared to around pH 10.0–10.5. Similarly, non‐alkaliphilic *Synechocystis* sp. *PCC 6803* was observed to lyse within 12 h at pH 11.3 (Zhang et al., 2009). These studies used batch cultivation. During batch cultivation, the cell concentration and pH increase with time, whereas nutrient concentrations and light availability decrease (Singh et al., 2014). This makes it hard to tell apart the effects of pH, illumination, and nutrient limitation. For example, cells may hoard trace metals at the start of the batch experiment when the pH is still low, so that they do not need to acquire any metals toward the end of the experiment when the pH is high. Also, even though the final pH might be high, most of the growth actually happened when the pH was still low. To overcome these limitations, here we used continuous cultivation of cyanobacteria in a chemostat. This involves continuously replacing spent culture with fresh medium at a stable ‘dilution’ rate. In a chemostat, over time a steady state will develop where growth can be studied at constant pH, nutrient concentrations and light availability.

We report the cultivation of cyanobacteria in chemostats at different pH values ranging from 10.2 to 11.4. We cultivated both a previously described alkaliphilic cyanobacterium Ca. *S. alkaliphilum* consortium (Ataeian et al., 2021) as well as a consortium of freshly sampled cyanobacteria from alkaline soda lakes. We show that biomass yields decline with pH, with a prominent role of photoinhibition at high pH. Above pH 11.4, bicarbonate uptake limitation prevented further growth of the tested haloalkaliphilic cyanobacteria.

## EXPERIMENTAL PROCEDURES

### Growth media

To mitigate the potential issues of volatilization and precipitation, reagents were introduced into the chemostat in two separate media, Medium A and B. Four versions of Medium A were formulated, each at a different pH (10.2, 10.5, 10.7, and 11.2). Medium A‐10.2 contained 199.50 mM Na_2_CO_3_, 101.00 mM NaHCO_3_, and 1.44 mM K_2_HPO_4_. Medium A‐10.5 contained 210.00 mM Na_2_CO_3_, 80.00 mM NaHCO_3_, and 1.44 mM K_2_HPO_4_. Medium A‐10.7 contained 230.00 mM Na_2_CO_3_, 39.68 mM NaHCO_3_, and 1.44 mM K_2_HPO_4_. Medium A‐11.2 contained 250.00 mM Na_2_CO_3_, 141.46 mM HCl, and 1.44 mM K_2_HPO_4_. Medium B contained 61.18 mM NaNO_3_, 18.32 mM NH_4_Cl, 40.87 mM MgSO_4_, 120.70 mM KCl, 3.42 mM CaCl_2_, 0.76 mM Ferric ammonium citrate, a 20 mL/L trace element solution. The latter contained: 500 mg/L Titriplex III (EDTA), 200 mg/L FeSO_4_·7H_2_O, 10 mg/L ZnSO_4_·7H_2_O, 3 mg/L MnCl_2_·4H_2_O, 30 mg/L H_3_BO_3_, 20 mg/L CoCl_2_·6H_2_O, 1 mg/L CuCl_2_·2H_2_O, 2 mg/L NiCl_2_·6H_2_O, 3 mg/L Na_2_MoO_4_·2H_2_O.

### Chemostat operation

Chemostats consisted of borosilicate glass bottles containing 1 L of medium and illuminated with LEDs as previously described (Haines & Strous, 2021). Photons were supplied at 60–350 μmol/m^2^/s as explained in the results and discussion section. All experiments were conducted at room temperature (20 ± 1°C, controlled by a central air conditioner) and maintained on a 16:8 h day–night cycle. 14.8 mL/h of Medium A and 0.52 mL/h of Medium B were provided continuously only during the daytime with a peristaltic pump and a syringe pump respectively. In total, 250 mL/day of fresh medium was added and an equal amount of spent media was collected each day via passive overflow, leading to a dilution rate of 0.25/day. Samples for analysis were collected at the end of each 16‐h light period and stored at −80°C until analysis.

### Cyanobacteria used and chemostat inoculation

Ca. *S. alkaliphilum*, an approximately 5 μm wide filamentous cyanobacterium (Figure S1 in Appendix S1), was previously enriched from alkaline soda lakes (Ataeian et al., 2021). Fresh microbial mats were obtained from Goodenough, Deer and Probe alkaline soda lakes (coordinates in Appendix S1: Table S1) in April 2023, homogenized in HDPE bottles (Nalgene, Thermo Scientific), transported to the lab and stored at 4–6°C. Before inoculation, equal volumes of homogenized mats from the three lakes were mixed together. For inoculation, The Ca. *S. alkaliphilum* consortium or homogenized mats were centrifuged in six falcon tubes (50 mL) for 15 min at 4500 × *g* to yield six pellets. These were washed three times with fresh medium. Two pellets were used to inoculate each of three (triplicate) chemostats. Next, 950 mL of Medium A and 50 mL of Medium B were added per bottle and a magnetic stirrer bar was introduced.

### Biomass and light intensity measurements

The optical density of chemostats was measured at 750 nm using a Thermo Scientific Evolution 60S photospectrometer. For ash‐free dry weight (AFDW, g/L), 0.1 L of the sample was filtered through a Whatman GF/F (0.7 μm pore size) fibreglass filter, that retained the filamentous cyanobacteria (Ataeian et al., 2019). The filter with wet biomass was then dried at 105°C for 16 h. The mass of this filter was recorded. Subsequently, the filter was ashed at 540°C for 4 h and the mass was measured again. The AFDW was calculated by dividing the difference in filter mass by the applied sample volume. The light at the centre of the chemostat bottles was measured using a submersible light metre (LI‐250A, LI‐COR Biosciences, USA).

### Alkalinity, pH and (bi)carbonate concentrations

CO_3_^2−^/HCO_3_^−^ alkalinity was diluted 201 times and measured using a Fisher Scientific Orion Star T910 pH Titrator with a standardized and certified 0.02 N H_2_SO_4_ titrant (Thermo Fisher Scientific FLSA2261). Dilution reduced the sample salinity, which affected the dissociation constant of bicarbonate (pK_2_) by reducing activity coefficients. A lower pK_2_ value (9.07) was used to calculate the actual CO_3_^2−^ and HCO_3_^−^ concentrations in the chemostats (Millero et al., 2006), as the original salinity of the medium used was 30.9 g/kg. The pH was measured by a pH metre (Thermo Scientific, VSTAR80) with a pH electrode suitable for alkaline solutions (Fisher Scientific, 8175BNWP). The daily bicarbonate consumption (mmol/day) was calculated by adding the culture's previous day's total bicarbonate content to the amount of bicarbonate introduced with the fresh medium, then subtracting the amount of bicarbonate lost with the effluent and the culture's current total bicarbonate content.

### Nutrient analysis

Nitrate was measured using ion chromatography (IC) with an anion‐exchange column (Dionex IonPac AS22; 4 × 250 mm; Thermo Scientific) after filtration of the sample with a syringe filter (0.2 μm pore size) and 100‐fold dilution with deionized water. Other samples for ammonium and trace elements analysis were filtered through a 0.7 μm pore size fibreglass filter (Whatman GF/F). The ammonium concentration was measured as described previously (Li et al., 2023). Major cations and trace elements were analysed using an Agilent 8800 Triple Quadrupole Inductively Coupled Plasma Mass Spectrometer (ICP‐QQQ, Agilent Technologies, Japan). We used glass beads and anionic detergent to disrupt the cell wall for trace elements determination in cells: 0.5 mL of culture was added to a lysing matrix tube (MP Biomedicals, Santa Ana, CA, USA), which contained 0.8 mL of 4% sodium dodecyl sulfate (0.1 mM EDTA, 0.25 mM NaCl, pH 7.2). The lysing tube was then processed three times in a bead mill homogenizer (Bead Ruptor 24, OMNI, USA) at speed 4.5 of 45 s. 5% HNO_3_ solution was added to lower the pH to below 2. Finally, the sample was diluted 100 times before being measured by the ICP‐QQQ.

### DNA extraction, sequencing, and analysis

DNA was extracted and quantified as described before (Li et al., 2023). Both 16S and 18S rRNA genes were amplified using a universal primer set (Yeh & Fuhrman, 2022). MiSeq sequencing was done as previously described (Sharp et al., 2017). Reads were processed using MetaAmp (Dong et al., 2017) to infer amplicon sequence variants (ASVs), relative abundance and diversity. Taxonomy classifications of ASVs were confirmed via comparison of the consensus sequences with GenBank using NCBI BLASTn.

### Protein extraction and metaproteomics

Methods for protein extraction, quantification, identification, and analysis have been previously described (Zorz et al., 2019). For microbial mats, based on the 16S rRNA amplicon sequences data, eight cyanobacterial strains were selected for creating two identification databases (Table S2 in Appendix S1). Nucleotide sequences of the corresponding strains were downloaded from NCBI. Subsequently, Metaerg 2.4.2 was used to predict open reading frames and the functions of encoded proteins (Dong & Strous, 2019). Highly similar proteins (>95% amino acid identity) were filtered by cd‐hit (Li & Godzik, 2006). Sequences of common contaminating proteins were added to the final database (https://www.thegpm.org/crap/).

## RESULTS AND DISCUSSION

Previous research used batch cultures to study the physiology of halo‐alkaliphilic cyanobacteria at high pH (Gao et al., 2023; Koch et al., 2022; Minagawa & Dann, 2023; Nies et al., 2017; Shipova Aleksandra et al., 2019). However, it is challenging to study microbial physiology while biomass density, light, pH, and nutrient concentrations are changing. Here, three sets of replicated (3x) chemostats were inoculated with Ca. *S. alkaliphilum* and associated heterotrophs. Each set was supplied with medium with a different pH: 10.2, 10.7 and 11.2 Although Ca. *S. alkaliphilum* was enriched from alkaline soda lakes and shown to grow at a pH above 11, it was unknown how its growth at high pH compared with other alkaliphilic cyanobacteria. Therefore, a fourth set of replicated chemostats was inoculated with fresh soda lake microbial mat samples, containing a large diversity of alkaliphilic cyanobacteria (this included Ca. *S. alkaliphilum*, which is naturally present in these mats). The medium supplied to this final set had a pH of 10.5 because our experiments with Ca. *S. alkaliphilum*, a pH of 10.5 resulted in the highest steady state pH value (see below).

Initially, the chemostats were not yet in a steady state. During this initial phase, growth was not yet limited by any external factor. The resulting growth rate was higher than the dilution rate, leading to gradual increases in pH and biomass, as well as decreases in light and nutrient concentrations, until a steady state was reached.

### Growth and limiting factors

**FIGURE 1:**
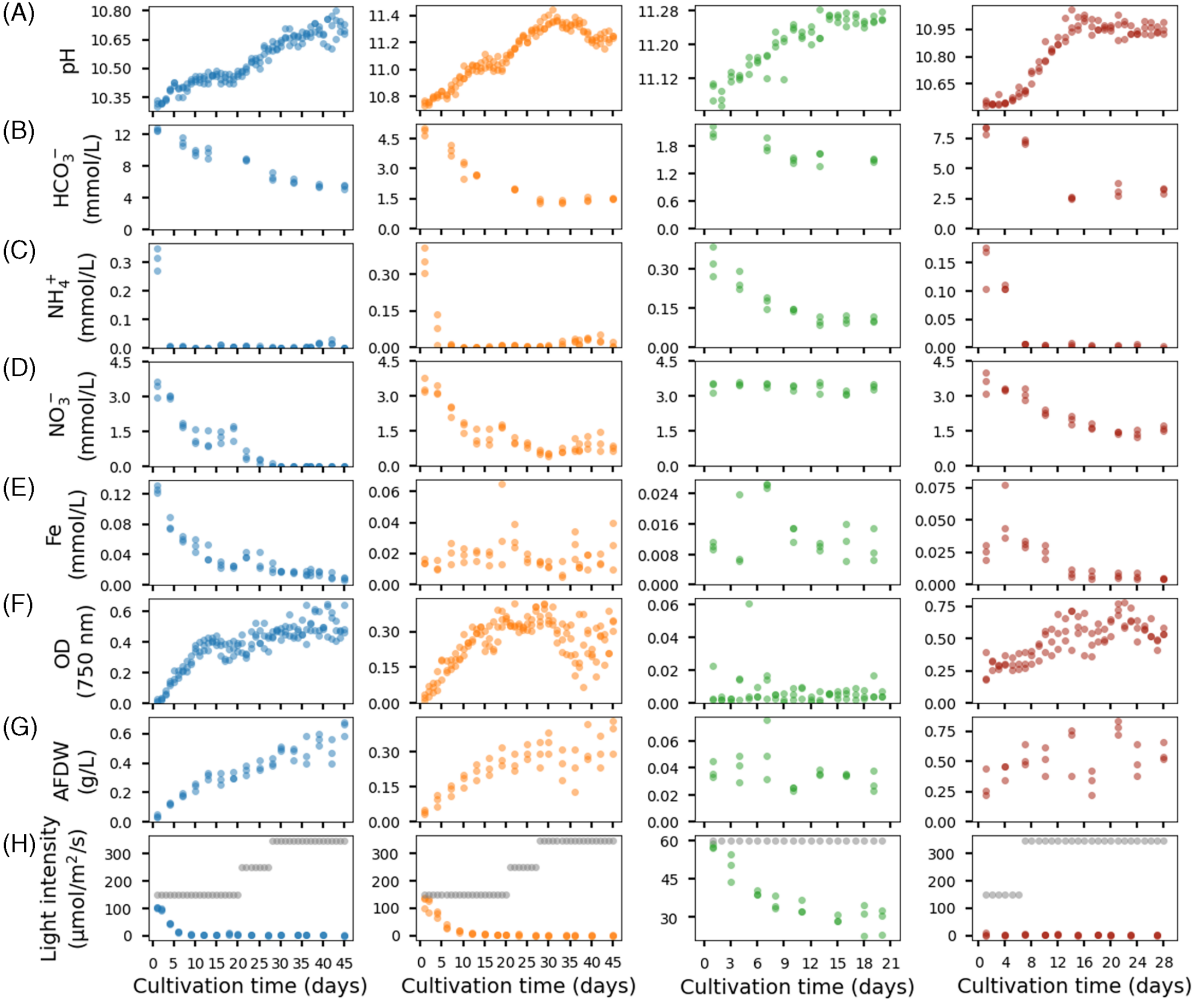
The dynamics of pH (A), HCO_3_^−^ (B), NH_4_^+^(C), NO_3_^−^ (D), Fe (E) concentrations, optical density (OD) (F), ash‐free dry weight (AFDW) (G), light provided (grey) and measured in the centre of the culture (H). The blue, orange, green and red scatters indicate the growth of Ca. *Sodalinema alkaliphilum* and associated heterotrophs fed in media at pH 10.2, 10.7 and 11.2, and fresh microbial mats fed with pH 10.5 medium, respectively.

In the Ca. *S. alkaliphilum* cultures fed with pH 10.2 medium, initially biomass and pH increased until a plateau was reached at pH 10.4 after 13 days (Figure 1, blue symbols). This pH increase was because of CO_2_ assimilation. From days 13 to 19, concentrations of biomass, bicarbonate, nitrate, and ammonium appeared stable, indicating a steady state was reached. Hence, a factor came to limit the growth of the cyanobacteria. No light was detected in the centre of the culture, indicating that light might be the limiting factor. To confirm this, we increased the provided light intensity from 150 to 250 μmol/m^2^/s on day 20. Over the subsequent 7 days (days 21–28), the pH increased from 10.4 to 10.6 and the biomass concentration increased. On day 28, no light was detected in the culture's centre and the concentration of nitrate had decreased to below the detection limit. To probe if light was again limiting growth, we increased the light intensity to 350 μmol/m^2^/s. This, however, did not result in additional growth (Figure 1, blue symbols). Apparently, the chemostats had reached a steady state with nitrate as the limiting substrate.

In the Ca. *S. alkaliphilum* cultures fed with pH 10.7 medium, biomass and pH increased during the initial 16 days, until the pH stabilized at approximately 11.1 (Figure 1, orange symbols). To test if light was limiting, light intensity was increased from 150 to 250 μmol/m^2^/s on day 20. This led to an additional increase in biomass and pH, and a decrease in nitrate until the pH reached 11.4 on day 28. When the light intensity was further increased to 350 μmol/m^2^/s, this did not lead to additional growth. Actually, a slight decline in growth was observed, as discussed below. This indicated that a factor other than light was limiting growth and it was not nitrate, as the nitrate concentration remained above 0.5 mmol/L.

At a pH as high as 11.4, the solubility of metals like Fe might be reduced. While Fe concentrations decreased between pH 10.3 and 10.7 (Figure 1, Appendix S2), they stabilized above pH 10.7, at 0.02 (±0.005) mmol/L (Figure 1E), not significantly different from the nitrate‐limited experiment of Figure 1A (*p* = 0.24). This was also true for other elements, such as Ca and Co (T‐test: *p >* 0.05). To further explore possible trace element limitations, we measured the concentrations of trace elements inside the cells after lysis (Appendix S2). If the uptake of trace elements would be limited, this might result in lower intracellular concentrations. On day 45, when the pH was 11.3, the intracellular Fe concentration was higher than measured at lower pH (10.7) in the experiment above (7.3 vs. 3.8 mg/kg, *p* = 0.006). The same trend applied to Ca and Co (*p >* 0.05). Although the Mn, Cu and Zn concentrations were slightly (21%–32%) lower than those in the pH 10.2 experiment (*p <* 0.05), differences remained relatively small, and it appeared that the availability of trace elements did not limit growth.

In the absence of light limitation and other limiting nutrients, we concluded that bicarbonate limited growth. During this experiment, the bicarbonate concentration decreased to 1.5 (±0.2) mM in the steady state (Figure 1, orange symbols, Appendix S3). This concentration was still high when compared with the published substrate affinity constants of bicarbonate transporters. These typically range from 15 to 120 μM for ATP‐dependent BCT1, ~95 μM for Na^+^ symporters BicA, and 2–5 μM for SbtA (Omata et al., 1999; Price et al., 2004; Shibata et al., 2002). However, these affinity constants were measured at a much lower pH (pH 9) and much lower CO_3_^2−^/HCO_3_^−^ ratio (0.21). For comparison, in our experiments, the CO_3_^2−^/HCO_3_^−^ ratios ranged between 25 and 192 (Figure S2 in Appendix S1). For the natronophilic cyanobacterium ‘*Euhalothece natronophila*’, the affinity constant (*K*_s_) for bicarbonate increased from 0.8 at pH 8.5 to 800 mM at pH 10.2 (Mikhodyuk et al., 2008). Thus, occurrence of bicarbonate limitation at 1.5 mM HCO_3_^−^ at pH 11.4 was consistent with previous measurements of bicarbonate affinity.

Increasing light intensity from 250 to 350 μmol/m^2^/s on day 28 led to a significant decrease in pH, and an increase in bicarbonate, ammonium, and nitrate concentrations, as well as a significant (*T*‐test: *p <* 0.001) decrease in optical density. This suggested the occurrence of photoinhibition, which might result from bicarbonate limitation. NADPH produced in photosynthesis might no longer be effectively used for CO_2_ fixation, leading to the accumulation of reactive oxygen species (Muramatsu & Hihara, 2012).

The negative effects of light were even more evident when we fed Ca. *S. alkaliphilum* with pH 11.2 medium. We initially provided a light intensity of 150 μmol/m^2^/s, similar to the other two experiments. However, in this case, we did not observe any increase in pH or biomass (data not shown). The lack of growth was not caused by the pH of 11.2 itself, as the pH in the previous experiment had already exceeded 11.2. However, when we restarted the experiment with a reduced light intensity of 60 μmol/m^2^/s, Ca. *S. alkaliphilum* was able to grow (Figure 1, green symbols). This showed that Ca. *S. alkaliphilum* is more light‐sensitive at high pH.

So far, we presented growth of Ca. *S. alkaliphilum* and associated heterotrophs at high pH levels, observing growth up to pH 11.4 with declining growth yields and increasing light sensitivity. To investigate whether other alkaliphilic cyanobacteria might display similar responses, we inoculated a new set of chemostats with a mixture of microbial mats freshly obtained from alkaline soda lakes. If those mats would harbour cyanobacteria that grow well at very high pH, those would be selectively enriched.

These chemostats were supplied with a pH 10.5 medium. To avoid light limitation, after 8 days of cultivation, the light intensity was increased from 150 to 350 μmol/m^2^/s. On day 13, the pH stabilized at 10.9 (Figure 1, red symbols) and the chemostats reached a steady state. With all nutrient concentrations higher than in the previous experiments, it was initially unclear what was the limiting nutrient. However, proteomics showed that these bacteria were not using nitrate, pointing at nitrogen limitation (see below). The culture's optical density and ash‐free‐dry‐weight showed fluctuations because of the frequent clogging of the effluent tubes caused by aggregated biomass. These specific consortia originated from microbial mats. As they were freshly collected, it was to be expected that they clumped together.

### Dynamics of microbial communities

**FIGURE 2:**
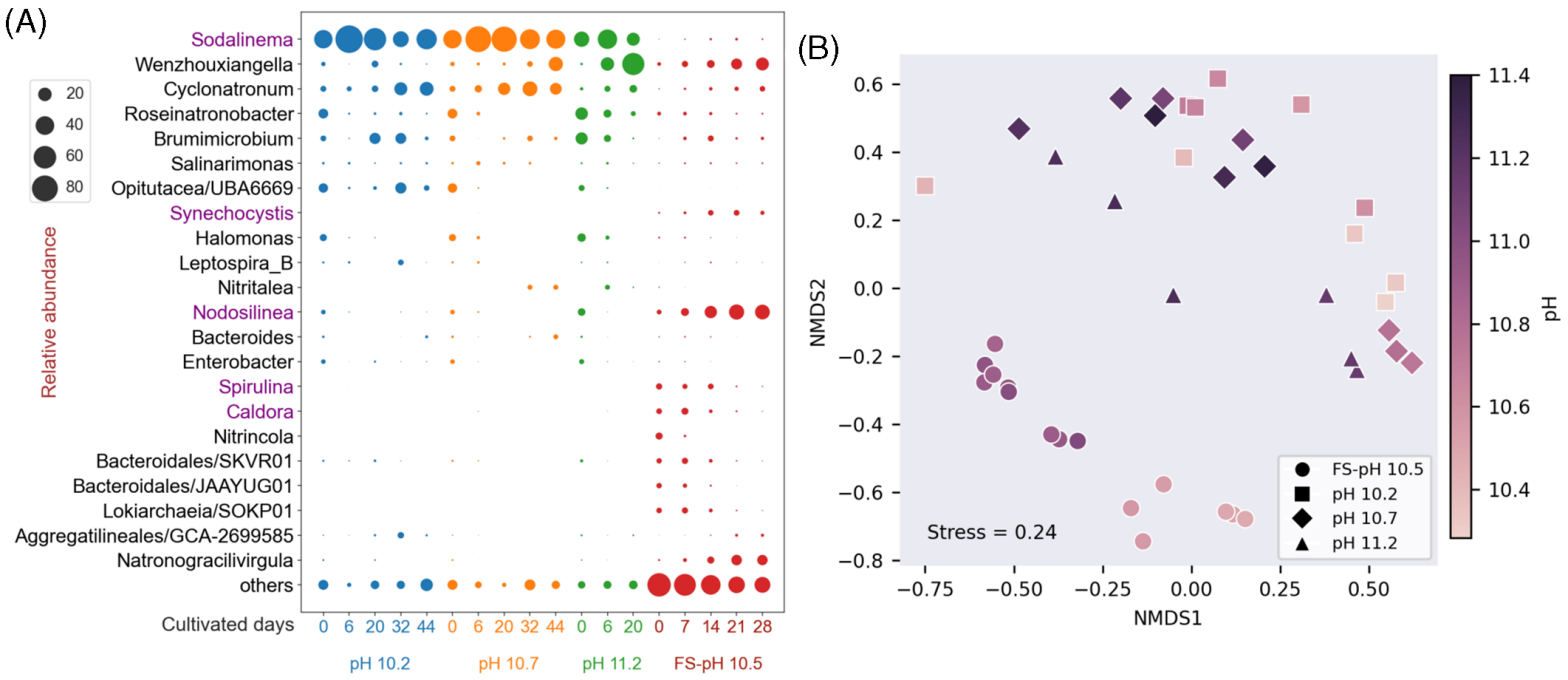
Microbial consortium dynamics. (A) Bubble plots showing the relative abundance of amplicon sequence variants (ASVs) at different time points. Data were aggregated by genus. Cyanobacteria are shown in purple. (B) Non‐metric multidimensional scaling (NMDS) plots using Bray–Curtis dissimilarity to visualize differences in community composition between experiments and time points.

Ca. *S. alkaliphilum*, an approximately 5 μm wide filamentous cyanobacterium (Figure S1 in Appendix S1) is not available in pure culture but grows as a consortium with associated heterotrophs (Ataeian et al., 2019). Therefore, we used 16S rRNA gene amplicon sequencing to study the effect of high pH on the makeup of the cyanobacterial consortium (Figure 2A, Appendix S4).

In each replicated chemostat experiment, the relative sequence abundance of Ca. *S. alkaliphilum* peaked shortly after the start of the experiment when growth was still exponential and not yet limited by nutrients or light. The maximum relative abundance observed for Ca. *S. alkaliphilum* was 90%, comparable to previous studies (Ataeian et al., 2019; Ataeian et al., 2021). During the subsequent periods of light or nutrient limitation, its relative abundance decreased to less than 50% in all Ca. *S. alkaliphilum* inoculated experiments. The consortium previously contained *S. alkaliphilum* as the only cyanobacterium. Here, we detected the presence of bacteria affiliated with *Nodosilinea* and *Caldora*. The relative abundance of *Nodosilinea* sequences was up to 7% on day 0 and decreased to ~0.1% in the following days. Similarly, the relative abundance of *Caldora* sequences was consistently up to 0.1%. As the abundance of *Sodalinema* decreased, we observed an increase in the relative abundance of *Cyclonatronum*, *Wenzhouxiangella*, *Brumimicrobium*, and *Opitutacea*. This could be explained by increased transfer of carbon from cyanobacteria to these heterotrophic bacteria at high pH. However, because the trend was only based on 16S rRNA gene amplicon sequencing data, other interpretations might also be possible. Therefore, the rise in the relative abundance of heterotrophic bacteria might suggest an increased transfer of carbon from Ca. *S. alkaliphilum* to its associated heterotrophs. This transfer might be most pronounced in the pH 11.2 experiment that experienced the strongest photoinhibition.

In the experiments with fresh microbial mats a very different, more diverse cyanobacterial consortium was enriched. Among cyanobacteria, a population affiliated with *Nodosilinea* (Figure S1 in Appendix S1) was most abundant, followed by *Spirulina*, *Caldora* and *Synechocystis. Nodosilinea* was previously identified as the most abundant cyanobacterium in the sampled microbial mats (Zorz et al., 2019) and in alkaline soda lakes in Kulunda, Russia (Samylina et al., 2014). *Nodosilinea* is a filamentous cyanobacterium with a width of approximately 1.5 μm. Bacteria that are abundant in nature do not always grow well in the laboratory, and this population of *Nodosilinea* was never cultivated previously. This indicated that these chemostats provided growth conditions more similar to the natural habitat than previous experimental setups (Haines et al., 2022; Sharp et al., 2017). The selective enrichment of *Nodosilinea* indicated that it outcompeted Ca. *S. alkaliphilum* in these chemostats.

Nonmetric Multidimensional Scaling (NMDS) analysis showed that the microbial communities of the *Nodosilinea* and the Ca. *S. alkaliphilum* consortia were distinct (Figure 2B). For both consortia, the community composition was mainly determined by either pH or cultivation time. These two factors could not be discriminated as in our experiments the pH always increased with cultivation time (Figure 1).

### Biomass yield

To compare growth physiology across experiments, carbon, and nitrogen consumption as well as biomass production rates were aggregated for chemostats that were in steady state (Table 1).

**TABLE 1:** The biomass yield of pH 10.2, pH 10.7 and FS‐pH 10.5 experiments.

Inoculum^a^	Light provided (μmol/m^2^/s)	Cultivated time (day)	Medium pH	End pH	N (mmol/day)	HCO_3_^−^(mmol/day)	Biomass (mmol C/day)	Yield (mol C/mol photons)
*Sodalinema*	350	30–45	10.2	10.7	0.99^A^	23.9^A^	5.5^A^	0.011^A^
*Sodalinema*	250	20–28	10.7	11.4	0.85^B^	9.6^C^	3.2^B^	0.006^C^
*Sodalinema*	350	29–45	10.7	11.2	0.76^C^	9.5^D^	3.4^B^	0.004^D^
Microbial mats	350	13–28	10.5	11.0	0.63^D^	19.2^B^	5.7^A^	0.009^B^

^a^ *Sodalinema*: the Ca. *S. alkaliphilum* and its associated heterotrophs; Microbial mats: the fresh soda lake microbial mat samples; Light provided: the provided light intensity; Medium pH: the pH of provided fresh medium; End pH: the culture pH in steady state; N: the nitrogen used in steady state; HCO_3_^−^: the bicarbonate used in steady state; Biomass: the average biomass produced in steady state based on AFDW; Yield: the HCO_3_^−^ consumption rate divided by two and further divided by the input energy in the form of light. Different uppercase letters in the same column indicate significant differences at the 0.05 confidence interval.

For each C‐mol of biomass growth, the consumption of two moles of bicarbonate was expected, because the carbon concentrating mechanism (CCM) first needs to convert bicarbonate into CO_2_ as follows: 2HCO_3_^−^ = CO_3_^2−^ + CO_2_ + H_2_O (Singh et al., 2014). However, after taking this factor into account, bicarbonate consumption was still much higher than observed biomass growth, measured as ash‐free dry weight (AFDW). This difference might be explained by the larger than expected amount of carbon that was transferred from cyanobacteria to heterotrophs in the steady state (Figure 2). To measure AFDW, we used a 0.7 μm pore size fibreglass filter, which retained large filamentous cyanobacteria (diameter 5 μm, Ataeian et al., 2019). Potentially, smaller heterotrophic consortium members may have passed through. Retention of the aggregated biomass in the experiments with fresh microbial mats was likely more complete, leading to higher AFDW measurements in that experiment. In addition, the AFDW measurements in that culture might have been overestimated due to the periodic clogging of the effluent tube. Therefore, bicarbonate consumption (mmol/day) was likely a better estimator for biomass yield than AFDW.

Biomass yield was consistently about 20% higher in the nitrogen‐limited cultures at pH 10.7 compared with the bicarbonate‐limited cultures at pH 11.4. In the absence of any limiting factor (days 3–12), the lower pH experiment also displayed fasted growth, based on both the accumulation of AFDW as well as bicarbonate consumption (*T*‐test: *p* < 0.05). In the fresh microbial mats experiment at pH 10.9, the biomass yield was in between that of nitrogen‐ and bicarbonate‐limited Ca. *S. alkaliphilum*. Biomass yield values were comparable with previous reports for Ca. *S. alkaliphilum* (Sharp et al., 2017).

### Protein expression

**FIGURE 3:**
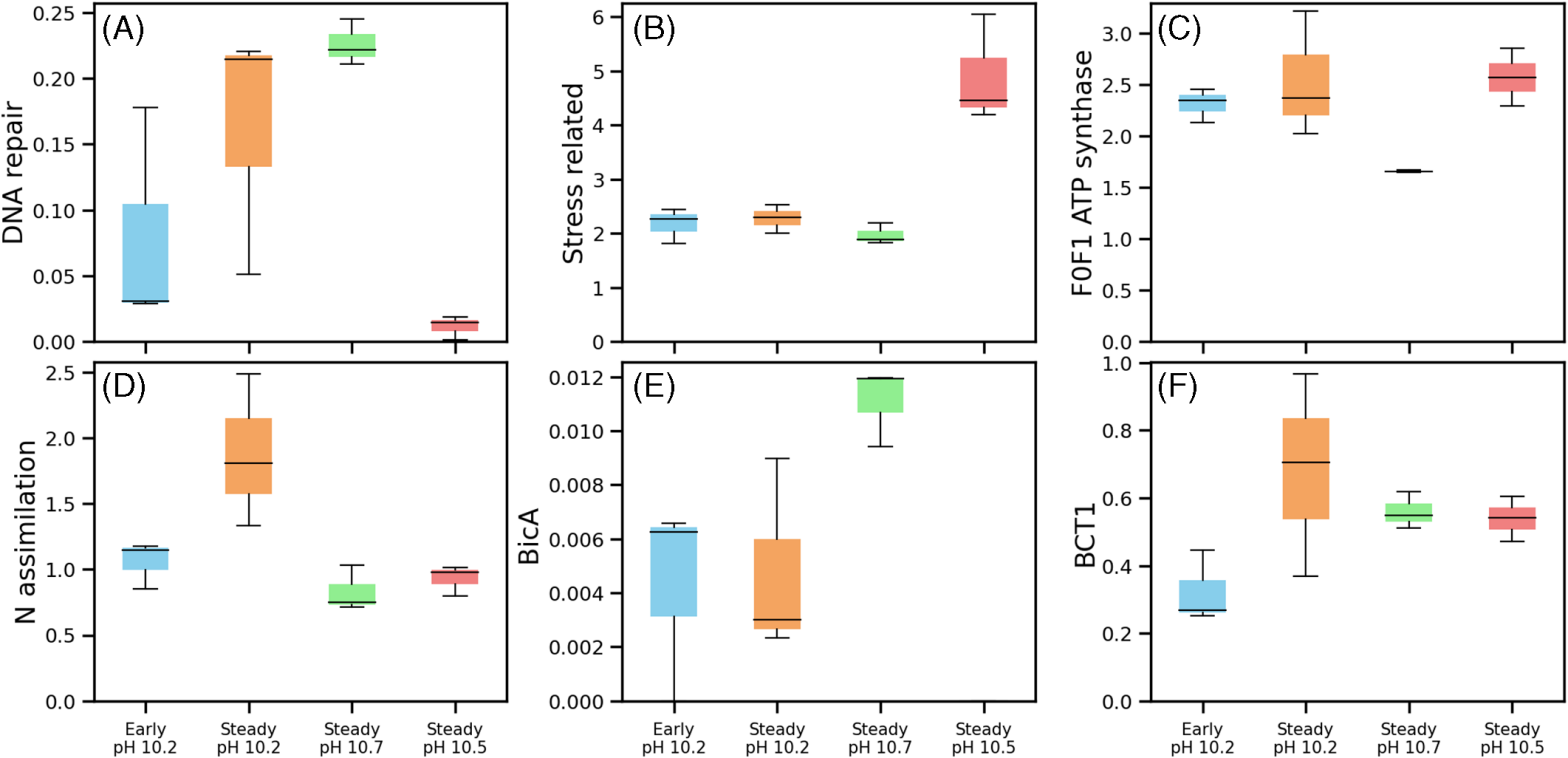
The protein expressions of Ca. *Sodalinema alkaliphilum* and *Nodosilinea* species. Blue and orange boxes indicated the early and steady states of samples collected from the chemostats fed with pH 10.2 medium, respectively. Green and red boxes indicated samples from the steady states of the chemostat fed with medium at pH 10.7 and 10.5, respectively. Each had three replicates.

At high pH, HCO_3_^−^ uptake might be a major challenge faced by cyanobacteria. Did Ca. *S. alkaliphilum* and *Nodosilinea* use the same mechanism for import of bicarbonate? To address this question, we performed proteomics (Figure 3). Proteomics was performed for exponential growth of Ca. *S. Alkaliphilum* at pH 10.4 (day 8), nitrogen‐limited Ca. *S. alkaliphilum at* pH 10.7 (day 35), and bicarbonate‐limited Ca. *S. alkaliphilum* at pH 11.3 (day 35). *Nodosilinea* was sampled in steady state at pH 10.9 (day 24).

Both Ca. *S. alkaliphilum* and *Nodosilinea* showed the expression of bicarbonate transporter proteins (Figure 3E, F). The expression level of the ATP‐dependent transporter BCT1 was two orders of magnitude higher than that of the Na^+^‐dependent transporter Bic. Expression of BCT1 increased with pH. No other types of bicarbonate transporters were detected in any of the samples. This suggested that BCT1 was the primary bicarbonate transporter in both species. Even though expression of Bic was low, it was still upregulated during bicarbonate limitation. These results were consistent with previous work (Ataeian et al., 2021).

Under bicarbonate‐limitation at pH 11.4, Ca. *S. alkaliphilum* exhibited significantly higher expression of proteins related to DNA repair compared with exponentially growing cells at pH 10.4 (Figure 3A). The expression of RadA (Zhou et al., 2006), RecN (Kil Yury et al., 2005), and DNA mismatch repair protein MutS (Drummond et al., 1997; Li, 2008) were elevated. The upregulation of these proteins suggested that Ca. *S. alkaliphilum* was coping with DNA damage under bicarbonate limitation. This may have been caused by production of free radicals during photosynthesis bottlenecked by a shortage of carbon dioxide (Takahashi & Murata, 2008), explaining the observed heightened sensitivity of the cyanobacteria to light at high pH.

In the nitrogen‐limited experiment, the expression of nitrogen assimilation proteins was higher compared with the bicarbonate‐limited experiments (*p* = 0.08) (Figure 3D). Nitrogen assimilation proteins included, for example, the P_II_ family nitrogen regulator (Forchhammer, 2008, 2010), the urea transporter, nitrogenase, and nitrate reductase. Interestingly, even in the nitrogen‐limited experiment, expression of nitrogenase remained very low. In previous studies, Ca. *S. alkaliphilum* always displayed high expression of nitrogenase, even in the presence of nitrate (Ataeian et al., 2021; Zorz et al., 2019). The lower nitrogenase expression may be a consequence of the strong selective forces in the chemostats (Gresham & Hong, 2015).

Interestingly, the *Nodosilinea* consortium did not express nitrate reductase. This might explain why this consortium did not consume nitrate. For the *Nodosilinea* consortium, we also observed that the expression of stress‐related proteins was high compared with for Ca. *S. alkaliphilum*. These proteins included universal stress proteins and molecular chaperones (Thirumalai & Lorimer, 2001), such as GroEL (Son et al., 2023) and GroES. Expression of universal stress proteins can be stimulated by conditions, such as nitrogen starvation, oxidative stress, and heat exposure (Vanbogelen et al., 1990). In our experiment, the temperature might have contributed to stress as the water temperature in the sampled lakes is below than the ambient temperature in our laboratory (20°C).Combining biomass production with the direct capture of CO_2_ from air could help make cyanobacterial biotechnology more sustainable. The higher the pH, the more effective the carbon capture will be, and the high pH may also prevent invasion of harmful organisms. Our study demonstrated cyanobacterial growth up to pH 11.4. However, this growth was associated with increased light sensitivity, potential DNA damage and lower biomass yield, especially above pH 11. For example, between pH 10.7 and 11.4, the biomass yield dropped by ~50%, which would translate into a doubling of the production costs and land needed per kg biomass (e.g., Gao et al., 2023; White & Ryan, 2015). Future work will show if other, even more alkaliphilic cyanobacteria exist that do not have these limitations, but they were not enriched from a biodiverse alkaline soda lake inoculum in our study.

## AUTHOR CONTRIBUTIONS

**Lianchun Yi:** Methodology; data curation; visualization; writing – review and editing; writing – original draft; funding acquisition; formal analysis; investigation; software; project administration; validation. **Ruchita Solanki:** Writing – review and editing; formal analysis; investigation. **Marc Strous:** Conceptualization; supervision; funding acquisition; writing – review and editing; resources.

## CONFLICT OF INTEREST STATEMENT

The authors declare no conflicts of interest.

## Supporting information


**Appendix S1.** Supporting Information.

**Appendix S2.** Supporting Information.

**Appendix S3.** Supporting Information.

**Appendix S4.** Supporting Information.

## Data Availability

Amplicon sequences can be found under the Bioproject PRJNA377096, Biosample SUB14485412. ASV sequences, read counts, and relative abundance data can be found at https://doi.org/10.6084/m9.figshare.25632279. Bicarbonate and carbonate calculation processes and results can be found at https://doi.org/10.6084/m9.figshare.25229750. Trace metal concentrations can be found at https://doi.org/10.6084/m9.figshare.25632330. The mass spectrometry proteomics data have been deposited to the ProteomeXchange Consortium via the PRIDE (Deutsch et al., 2023) partner repository with the dataset identifier PXD052563.
