# Supplementary material for: In search of the pH limit of growth in halo‐alkaliphilic cyanobacteria
Source: Environ Microbiol Rep. 2024 Aug 11;16(4):e13323. doi: 10.1111/1758-2229.13323 (PMC11317126; doi:10.1111/1758-2229.13323)
Supplement: Supplementary file 1 — Appendix S1. Supporting Information. [file EMI4-16-e13323-s001.docx]

**Figure and Tables**

**Captions**

**Figure S1** Fluorescent microscopic images of *Ca. S. alkaliphilum* and *Nodosilinea*.

**Figure S2** The dynamic of CO_3_^2-^/ HCO_3_^-^ ratios.

**Table S1** The coordinates of microbial mat samples.

**Table S2** NCBI accession numbers of two proteome identification databases.

**
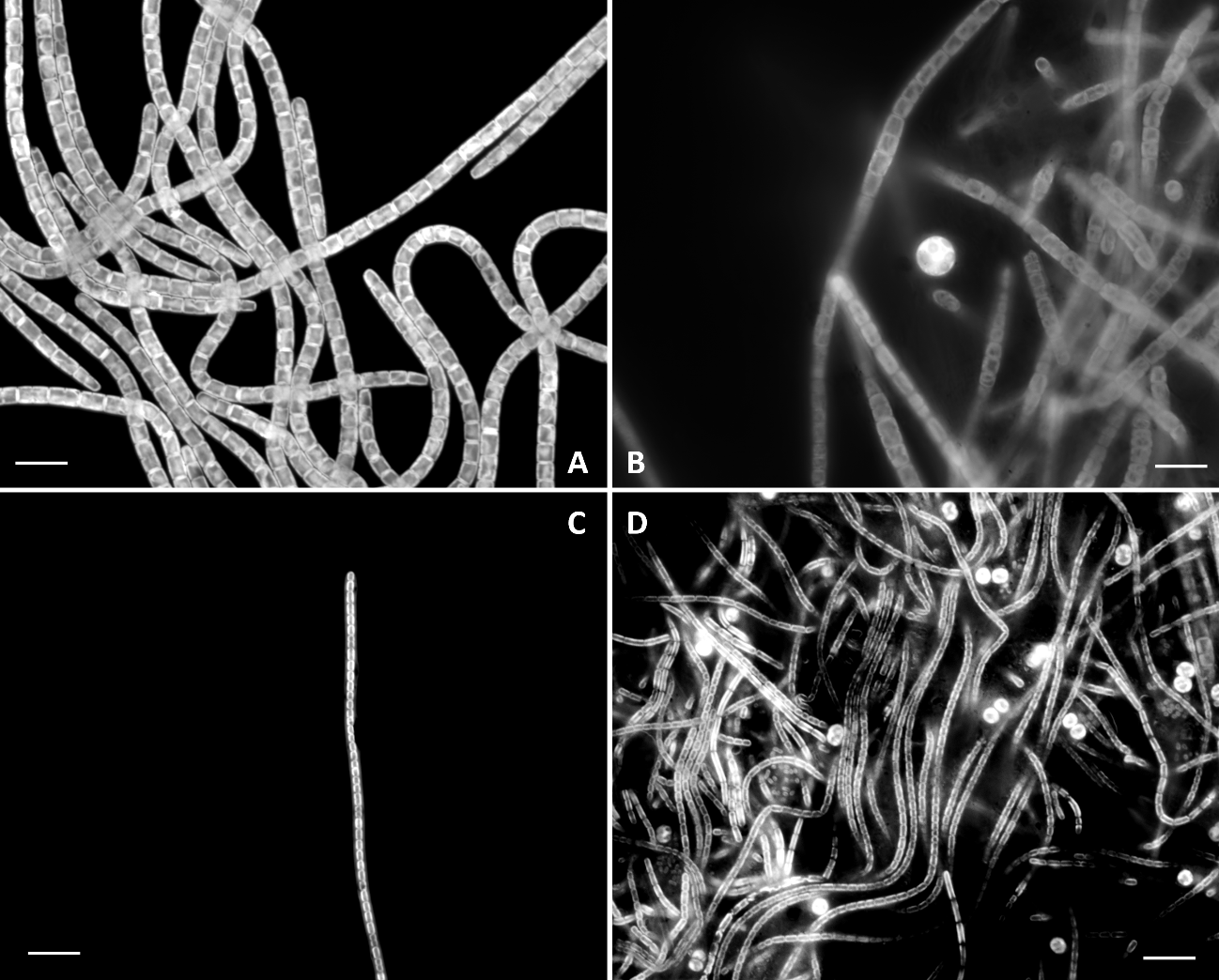
**

**Figure S1** Fluorescent microscopic images of *Ca. S. alkaliphilum* and *Nodosilinea*. (A-B) The *Ca. S. alkaliphilum* microbial consortium. (C-D) The *Nodosilinea* microbial consortium. Scale bars: 10 μm.


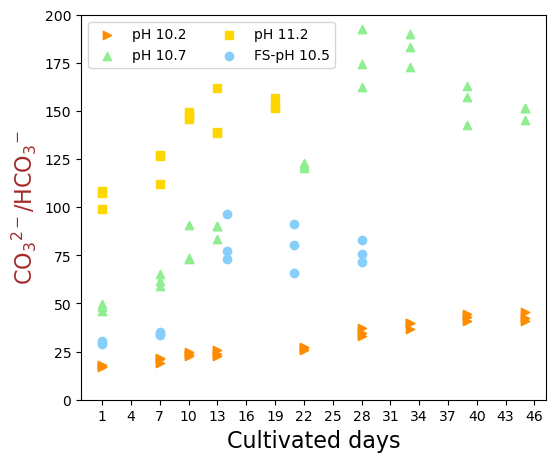


**Figure S2** The dynamics of CO_3_^2-^/ HCO_3_^-^ ratios. Orange and green indicated the cyanobacteria growth in media with pH 10.2 and 10.7 respectively, yellow represented the experiment using pH 11.2 medium to grow cyanobacteria. Blue showed the fresh microbial mats fed with pH 10.5 medium experiment. Each experiment had three biological replicates

**Table S1** The coordinates of microbial mat samples.

| **Lake** | Goodenough | Deer | Probe |
| --- | --- | --- | --- |
| **Latitude** | 51.33 N | 51.35 N | 51.45 N |
| **Longitude** | 121.64 W | 121.25 W | 121.39 W |

**Table S2** NCBI accession numbers of two proteome identification databases.

| **Inoculum** | **Medium pH** | | | **Sample pH** | **Sampling day** | **Limiting substrate** | **Identification database** |
| --- | --- | --- | --- | --- | --- | --- | --- |
| **Sodalinema** | | 10.2 | 10.4 | | Day 8 | - | GCA_007693465.1 |
| **Sodalinema** | | 10.2 | 10.7 | | Day 35 | Nitrogen | GCA_007693465.1 |
| **Sodalinema** | | 10.7 | 11.3 | | Day 35 | Bicarbonate | GCA_007693465.1 |
| **Microbial mats** | | 10.5 | 10.9 | | Day 24 | Unknown | GCF_003017855  GCA_018970085  GCA_016403105.1  GCF_014697375.1  GCA_012911975.1  GCA_003249105  GCA_000763385.1 |
